# Toxicological Risks of the Cobalt–Chromium Alloys in Dentistry: A Systematic Review

**DOI:** 10.3390/ma15175801

**Published:** 2022-08-23

**Authors:** Brigitte Grosgogeat, Alina Vaicelyte, Rémy Gauthier, Christine Janssen, Marc Le Borgne

**Affiliations:** 1 Laboratoire des Multimatériaux et des Interfaces, UMR CNRS 5615, Université Claude Bernard Lyon 1, Univ Lyon, 69008 Lyon, France; 2 Hospices Civils de Lyon, Service d′Odontologie, 69007 Lyon, France; 3 Faculté d′Odontologie, Université Claude Bernard Lyon 1, Univ Lyon, 69008 Lyon, France; 4 CNRS, INSA de Lyon, UCBL, MATEIS UMR CNRS 5510, Lyon, Bât. Saint Exupéry, 23 Av. Jean Capelle, 69621 Villeurbanne, France; 5 Institut de Formation en Masso-Kinésithérapie pour Déficients de la Vue (IFMK DV), 69373 Lyon, France; 6 Small Molecules for Biological Targets Team, Centre de Recherche en Cancérologie de Lyon, Centre Léon Bérard, CNRS 5286, INSERM 1052, Université Claude Bernard Lyon 1, Univ Lyon, 69373 Lyon, France

**Keywords:** cobalt, cobalt–chromium alloys, dental, mucosa, oral, biocompatibility, type IV hypersensitivity reaction, toxicity

## Abstract

***Background:*** The toxicological risk of Co-Cr dental alloys is actually a sensitive subject with the European regulatory changes, namely regulation (EU) 2017/745 and annex VI to the CLP regulation (EC) 1972/2008. ***Objectives***: The objective of this review is to conduct a rigorous analysis of the cytocompatibility of cobalt–chromium (Co-Cr) dental alloys. Considering various parameters such as cytotoxicity, type IV hypersensitivity reaction, sensitization, and irritation, we investigated evidence of toxicity of Co-Cr in human dental applications. ***Data sources***: Specific search strategies were performed in three electronic databases, namely *Medline*, *Embase*, and *Web of Science*, using a main restriction in the search regarding the publication date (1995–2022). ***Study selection***: Out of a total of 836 articles, only 21 studies were selected and analyzed according to PRISMA methodology. ***Results***: Among them, 10 in vitro studies using human samples and 11 in vivo studies on human patients were distinguished. Most of the in vitro studies confirmed that Co-Cr alloys have a good cytocompatibility compared to Ni alloys. Regarding the in vivo studies, it appeared that Co-Cr could rarely cause sensitization, irritation, and allergic reactions. Reactions were mainly observed for people allergic to Co or Cr. Nevertheless, titanium-based materials showed better results. ***Conclusions***: This study proposes a new state of the art on Co-Cr dental alloys and will thus be very useful for carrying out additional studies. ***Relevance***: This review will help practitioners in their daily clinical choice.

## 1. Introduction

In dentistry, cobalt–chromium (Co-Cr) alloys have been used for a long period of time. While they mainly contain Co and Cr, other metals such as manganese (Mn), molybdenum (Mo), or nickel (Ni) are also present [1]. For patients who have lost teeth, these Co-Cr alloys are a common material for removable dental prostheses. These applications are designed to replace missing teeth with artificial teeth supported by a metal alloy [2]. Co-Cr is relatively cheap, features good strength and stiffness, and shows suitable longevity. All of these qualities make it a good material for dental prosthesis production. These alloys are also widely used for orthodontics with brackets, arch wires, and bands.

Co-Cr alloys are also known for their good cytocompatibility parameters (e.g., passivation layer) [3], but several risk factors remain involved. The primary concerns are a resulting increased sensitization, allergic reaction, and oral cavity inflammation. It is hence important to analyze the toxicological data of Co-Cr alloys, especially bearing in mind that the use of medical Co-Cr alloys has been questioned in recent years. In 2020, there was a radical change around the regulation of metallic Co, which in Europe has become a carcinogenic, mutagenic, or toxic-for-reproduction (CMR) substance [4]. It now belongs to category 1B of CMR substances (carcinogenic 1B, mutagenic 2, toxic for reproduction 1B), and its use is limited to a specific concentration of 0.1% in the final product. With a Co concentration lying between 35 and 65%, this has a direct impact on the application of Co-Cr dental alloys [4].

It has been shown that Co-Cr alloys induce low irritation and sensitization levels, ensuring low risks for allergic reactions [1]. Various pure or base metal alloys still have risks of causing allergic reactions in the oral cavity. They can also induce non-adequate immune responses. One of the most common side effects of using these prosthetics is the increased sensitization [5]. Biological responses to dental alloys are affected by the susceptibility of metals to corrosion and ionic release. Although usually metal ions might be washed away by saliva, the removable prostheses are used for a longer period of time with close contact to the oral cavity, decreasing the organism’s capacity to ensure its physiological functions. That is especially evident with an upper jaw prosthesis, where the palate might not come in contact with saliva. As a result, released metal ions will congregate under the metal frame, inducing local irritation and further oral cavity problems [2].

Before investigating the potential risks of placing metal frames in the oral cavity, it is important to assess the cytocompatibility of each Co-Cr alloy used in human dental applications. The aim of the review is to carry out a systematic analysis of the literature regarding the specific criteria of cytocompatibility and biocompatibility. For this, various parameters will be analyzed in the selected articles, such as cytotoxicity, allergic reaction, sensitization, and irritation.

## 2. Methods

### 2.1. Data Sources and Search Strategy

This review was prepared according to the PRISMA statement [6]. Three databases, namely *PubMed*, *Embase*, and *Web of Science*, were systematically screened. The references cited in the articles were also checked and could be included in the search.

Search strategy was developed according to ISO 7405:2018 (evaluation of cytocompatibility of medical devices used in dentistry) and performed in these selected electronic databases. The search terms were divided in three parts: (i) CoCr alloy, for example, cobalt-chromium, Co-Cr, and CoCr; (ii) toxicity, for example, cytocompatibility, toxicity, cytotoxicity, sensitization, allergy, dermatitis, irritation, and genotoxicity; and (iii) dental fields, for example, dentistry, dental, prosthodontic, mucosa, periodontics, saliva, enamel, and oral. Then, this systematic review combined search terms and synonyms related to toxicological risks, including ((((“Cobalt-chromium” OR “Co-Cr” OR “CoCr”) AND (Biocompatib* OR Toxic* OR Cytotoxic* OR Sensitiz* OR Allerg* OR Dermatitis OR Irritat* OR Genotoxic* OR Gene*)) NOT (orthopaedic OR hip OR valvular OR leg OR limb)) AND ((((Dental) OR (Dentist*)) OR Prostho*)) OR (Mucosa OR Periodo* OR Saliv* OR Enamel OR Oral))) OR ((((((Toxicity Tests[MeSH Terms]) OR (Mutagenicity Tests[MeSH Terms])) OR (Dermatitis[MeSH Terms])) OR (Hypersensitivity[MeSH Terms])) AND (chromium alloys[MeSH Terms])) and (Dentistry[MeSH Terms])).

Articles identified using the search strategy were imported to COVIDENCE systematic review software (Covidence, Melbourne, Australia) [7] to import references and view duplicates, titles, abstracts, and full texts.

### 2.2. Study Selection

The titles and abstracts of all articles identified by the electronic search were read and assessed by two authors (A.V. and B.G.) until March 2022. All titles and abstracts were examined and selected in accordance with the eligibility criteria. Those that appeared to fulfill the inclusion criteria or with insufficient data in the title and the abstract were selected for full analysis. The two authors were also the two reviewers and independently assessed the full-text articles. Any disagreement on the eligibility of studies included was resolved through discussion and consensus.

One author (A.V.) extracted the data using a pre-piloted data collection form, and a second author (B.G.) verified data extraction independently for completeness and accuracy. Data obtained were as follows: general study details (first author, date of publication), type (in vitro/in vivo), population or sample size, criteria, methods, and outcomes of studies. Any potential conflict was resolved by a joint discussion between the two authors.

### 2.3. Inclusion and Exclusion Criteria

All research articles that have evaluated the toxicological risks of Co-Cr dental alloys in humans or human samples were eligible for inclusion. More precisely, any clinical study in humans or in vitro study on human samples or cells evaluating the cytocompatibility of Co-Cr alloys used in dentistry was selected. Then, the published scientific articles from January 1995 to March 2022 were systematically assessed for this review. 

All studies involving non-dental procedures (such as orthopedic, valve, breast, or limb prostheses) and those not dealing with Co-Cr alloys were withdrawn. Unreliable studies were removed, along with duplicate or overlapping data. The search strategy was conducted to exclude abstracts, literature review articles, conferences and commentary articles, editorials, books, theses, articles without available full text, and publications older than January 1995.

The set of inclusion and exclusion criteria were established by consensus of all the authors after discussion while considering the research question and the objectives of the study.

### 2.4. Data Extraction and Study Quality Assessment

The risk of bias was assessed according to the GRADE-tool adaptation [8]. As the current systematic review deals with both in vitro and in vivo, one risk assessment was performed for each type. Regarding the in vitro studies, the process was adapted following two previous systematic reviews based on in vitro studies [9,10] because there is no known assessment method for this kind of study. Two authors (B.G. and R.G.) categorized the articles as “high”, “moderate”, “low”, or “very low” level of evidence. The two authors read and grade separately the different studies according to 5 criteria: study limitations, inconsistency, indirectness, imprecision, and publication bias, as defined in the GRADE-tool adaptation [8]. Then, B.G. and R.G. discussed together about their results. Disagreements were resolved by discussion to reach consensus. Precision about the results are provided within the results section. 

## 3. Results

### 3.1. Study Selection

**Figure 1:**
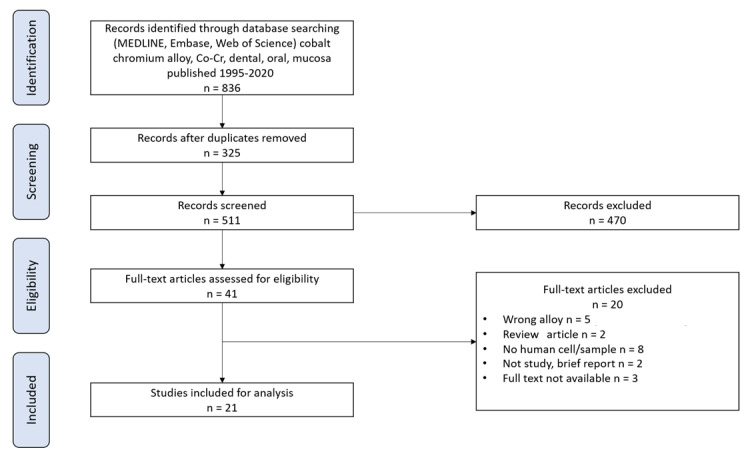
Flow diagram of study selection.

The initial electronic search using the keyword combination returned 836 articles (Figure 1). After the removal of duplicates, 511 records were obtained. Then, titles and abstracts of these 511 articles were screened, and 470 articles were excluded because they were irrelevant in regard to the inclusion criteria. A selection of 38 articles were fully read after removing 3 more articles (full text not available) [11,12,13]. A consensus between the two readers (A.V. and B.G.) was reached to determine which studies fully fulfilled the selection criteria. Then, 17 papers were excluded [14,15,16,17,18,19,20,21,22,23,24,25,26,27,28,29,30], resulting in the inclusion of 21 individual studies (Table 1, Figure 1).

A major inconsistency defined during the GRADE assessment of the in vivo studies lies in the analysis of dental technicians rather than patient wearing metal alloys in the oral cavity. Furthermore, case studies including a single patient were considered to lead to inaccuracies in interpretation (Könönen et al. [31], Song et al. [35], Table 2). 

For in vitro studies, limitations were stated when no quantitative data were provided. Indirectness was considered when Co-Cr was not compared to another material, and imprecisions were considered for low number of samples (< 5) or no information about the sample number (Table 3).

### 3.2. Cytotoxicity of Co-Cr Alloys Based on In Vitro Studies

Ganbold et al. [48] conducted an in vitro experiment in order to assess human adipose-derived stem cell (hADSC) behavior on a three-dimensional (3D)-printed Co-Cr alloys in comparison to a Ni-Cr alloy. Cell morphology was examined by a field emission scanning electron microscope, cell proliferation with a bromodeoxyuridine assay kit, and cell viability with a water-soluble tetrazolium salt assay kit. The Ni-Cr alloy was associated with significantly lower cell proliferation and viability in comparison to the Co-Cr ones. Proliferation for the Ni-Cr group presented an OD (optical density) value of 0.23. For all Co-Cr alloys, OD values were higher (0.38 for casting group, 0.33 for milling group, and 0.42 for 3D group). It revealed that Co-Cr alloys are more cytocompatible than Ni-Cr alloys. Comăneanu et al. [45] examined cytocompatibility of different Ni-Cr (N1, N2, N3) and Co-Cr (C1, C2, C3) alloys. While all materials exhibited moderate to high cytocompatibility, higher content of Co-Cr was associated with better cell adhesion. Cytocompatibility of alloys was summarized in the following descending order: C1 > C3 > N2 > N3 > C2 > N1. 

Forster et al. [42] and Gălăţeanu et al. [46] observed that Co-Cr exhibited good cell proliferation. Forster et al. [42] studied attachment and proliferation rate of cultured human epithelial cells on polished lithium-disilicate, yttrium-modified zirconium dioxide, and Co-Cr alloys. Cell attachment (24 h) and proliferation (72 h) were investigated using MTT (3-(4,5-dimethylthiazol-2-yl)-2,5-diphenyltetrazolium bromide) and AlamarBlue^®^ assays. All surfaces exhibited significant cell proliferation in comparison to the control group. Li-disilicate and zirconia exhibited the highest and lowest cell attachment, respectively. It revealed that all restorative materials, including Co-Cr, were equally suitable for subgingival restorations, but Li-disilicate had the best cytocompatibility. Although Co-Cr did not have the highest preferable parameters, it was still within the range. Regarding the study of Gălăţeanu et al. [46], they examined electrochemical behavior of two Co-Cr dental alloys (Wirobond 280 and Wirobond C also containing gallium and Mn). Their electrochemical aspects were examined in the artificial Erikson saliva at temperatures between 25 and 55 °C by potentiodynamic polarization and electrochemical impedance spectroscopy. Results showed that Wirobond 280 exhibited a greater cell viability and was associated with smaller levels of intracellular reactive oxygen species (ROS).

McGinley et al. [39] tested cytocompatibility and effect of Ni-Cr alloys on human-derived oral mucosa. Cytocompatibility was assessed by histological analysis for cell viability parameters, inflammatory cytokine expression, oxidative stress responses, and cellular toxicity. Co-Cr had significantly better cytocompatibility than the Ni-Cr alloy. Oral mucosal models treated by Ni-Cr alloy were associated with (i) significant reductions in cell viability and (ii) increases in oxidative stress, inflammatory cytokine expression, and cellular toxicity (in comparison to untreated oral mucosal models). With a continuous 72-hour observation, the higher Ni levels in the alloys generated the higher toxicity. In the following year, McGinley et al. [40] also provided more supportive evidence for Co-Cr better cytocompatibility. In this new study, each alloy was exposed to a 3D human-derived oral mucosal model for 2–72 h. Immersion solutions of Ni-Cr base-metal alloy showed significantly lower cytocompatibility than Co-Cr alloys. In comparison to controls, Ni alloy was associated with significantly decreased cell viability, increased oxidative stress, inflammatory cytokine expression, and cellular toxicity levels. The Co-Cr alloy did not increase oxidative stress or cellular toxicity when compared to controls. These findings directly supported the good Co-Cr cytocompatibility as opposed to Ni-Cr.

Good Co-Cr cytocompatibility was also confirmed by Puskar et al. [44]. The aim of the study was to determine the cytotoxicity of the direct metal laser-sintered (DMLS) and cast Co-Cr-Mo dental alloy on human MRC-5 fibroblast cells. This in vitro study suggested that Co-Cr-Mo alloy did not have a cytotoxic effect and could be used for application in dentistry. Cytotoxic effect was not observed in either conventionally cast Co-Cr-Mo alloys. In another in vitro study conducted by Rusu et al. [41], the cytotoxicity of Ni-Cr and the Co-Cr alloys was studied on pure cell line dermal fibroblasts and of those obtained from skin biopsies. The corresponding results highlighted their non-cytotoxic effect. For example, after 7 days of inoculation, the cells did not detach from the plate and then grew well in contact with both alloys.

On the other hand, an in vitro analysis of the effects of Co-Cr alloys on human gingival fibroblasts (HGF) and osteoblasts by Kim et al. [50] provided evidence that Co-Cr alloys might exhibit cytotoxicity. Cytotoxic and inflammatory effects of Co-Cr alloys were investigated through the activation of NF-E2-related factor 2 (Nrf2)/antioxidant response element (ARE). The alloys were revealed to be cytotoxic to HGF and osteoblasts. It significantly increased ROS production, upregulated pro-inflammatory cytokines, and increased levels of inflammatory mediators (iNOS-derived nitrite oxide and COX-2-derived prostaglandin E2).

According to Imirzalioglu et al. [36], cytotoxicity of alloys was affected by recasting. The effect of repeated casting on gingival fibroblast cytotoxicity was analyzed by an in vitro study. Three disks were selected, namely high noble gold–platinum (Au-Pt, *n* = 60) alloy and two base metal alloys (Ni-Cr and Cr-Co, *n* = 20). Cytotoxic effects were examined on human gingival fibroblast with a MTT colorimetric assay. Recasting significantly increased ionic release in both Co-Cr and Ni-Cr alloys. Ni-Cr alloys were associated with higher cytotoxicity, especially after recasting Ni-Cr alloys. Other factors such as composition must therefore be considered. 

In conclusion, in vitro studies showed that Co-Cr alloys have good cytocompatibility, which is highlighted by cell adhesion and proliferation as well as a non-cytotoxic effect. However, there are some exceptions. Alloy recasting may increase elemental release in Co-Cr alloys associated with higher cytotoxicity.

### 3.3. Cytotoxicity of Co-Cr Alloys Based on in Vivo Studies

Seldén et al. [32] measured the effect of cobalt chromium molybdenum (Co-Cr-Mo) exposure to lung disorders. The 37 participants were dental technicians with at least 5 y of exposure to dust from Co-Cr-Mo alloys. All participants agreed to undergo radiography to examine their lung condition. Aligning with previous studies that have shown toxic Co-Cr effects, Seldén et al. concluded that the dust from Co-Cr-Mo dental constructions can cause pneumoconiosis. Six participants in total exhibited radiological parameters associated with Co-Cr-Mo alloys. Additionally, the authors found that subjects from an environment with local exhaust ventilation showed better results at the end of the study. In a follow-up study, Seldén et al. [33] investigated the effect of Co-Cr-Mo exposure to lung disorders for three patients with confirmed pneumoconiosis cases in dental technicians. Pneumoconiosis was associated with inorganic dusts resulting from the handling of Co-Cr-Mo dental alloys. Such results are important to analyze because it would reveal toxic Co-Cr effects. However, the primary causes for the reported cases cannot be exclusively reduced to alloys because patients already had lung problems. Moreover, the scope of these studies was not comprehensive enough to draw conclusions. We cannot dismiss the fact that the study is 25 years old, as the results are strongly influenced by the lack of local exhaust ventilation in laboratories. Nowadays, local exhaust ventilation is mandatory in all laboratories. 

In a recent study, Yu et al. [47] examined the in vivo biocompatibility of four different crown materials, namely Co-Cr, Au-Pt, titanium (Ti), and zirconium (Zr). Twelve months after use (*n* = 196), probing depth (PD) and gingival crevicular fluid (GCF) volumes for all groups were significantly higher when compared to the control group. Zr- and Ti-based materials showed the best results. The Ti group had the highest concentration of osteoprotegerin (OPG), and Ti and Zr groups had smallest concentrations of receptor activator of nuclear factor kappa-Β ligand (RANKL) and calcium ion as well as the smallest RANKL/OPG ratio. In conclusion, the Co-Cr biocompatibility was considered as poor. 

Thus far, various studies have provided evidence that Ti is preferred over Co and Ni in terms of cytotoxicity, and the Ni exhibited the least-suitable parameters. Martín-Cameán et al. [43] compared the ionic release of aluminum (Al), copper (Cu), Cr, Mn, Ni, Ti, and vanadium (V) in oral mucosa cells from dental implants. The patients wore conventional orthodontic appliances (brackets, arch wires, and bands) and were additionally treated with mini-screws. A control group was added to the study. Few released traces of Co and V were observed. For other metals, the following order has been established: Cr < Ni < Ti < Cu < Al. Significant differences in comparison to the control group were observed in Ni release for orthodontic and orthodontic + mini-screw groups as well as in Cu release for the orthodontic group. However, mini-screws alone were not associated with a significant increase of metal release in all cases. These results suggested that Co, Cr, and Ni were released faster than Ti.

In a 5-year in vivo study, Baričević et al. [37] observed the genotoxicity of Co-Cr-Mo and Ni-Cr alloys when exposed to contact with oral cavity. Genotoxicity was examined using alkaline comet assay, and the cell viability was assessed with trypan blue exclusion test on 30 patients wearing prosthodontic appliances and 25 controls. Comet assay parameters (tail length and percentage DNA in the tail) were significantly higher in the group wearing prosthodontic appliances (both Co-Cr-Mo and Ni-Cr alloys) in comparison to the control group. The mean of tail length was 13.13 for the control group and 15.85 for the group wearing prosthodontic appliances, independently of the material used, while the percentage DNA in the tail was 0.36 for the control group and 2.07 for the experimental group. The results showed that metal ions released by both alloys could cause DNA damage of oral mucosa cells.

A later study by Katsoulis et al. [34] led to quite different conclusions. The research was specifically designed to assess the effects of using Ti in removable partial dentures (RPDs) of the Ti6A17Nb-alloy for 10 patients. RPDs were produced from Co-Cr alloy (Remanium GM 800+) and Ti6A17Nb alloy (Girotan L) for comparison purposes. Patients completed a questionnaire entitled VAS (visual analogue scale) after 1, 3, and 6 months for each RPD. After 6 months, significant biological differences between both alloys were not observed, and patients did not report toxic effects. It means that the Ti6A17Nb-alloy for RPDs could be regarded as equivalent to RPDs in Co-Cr. Hence, it suggests that Co-Cr-alloy was successfully applied at least during 6 months after the installation of RPDs.

In vivo studies provide more controversial results. In some cases, Co-Cr alloys can release metal ions, leading to cytotoxic effects to human oral mucosa. 

### 3.4. Sensitization and Irritation to Co-Cr Alloys

Könönen et al. [31] performed a 2-year clinical report aimed at investigating the effects of Ti-RPDs. It was previously reported that Co, Ni, and Cr caused hypersensitivity and in some cases gingivitis and stomatitis. Based on this evidence, it was presumed that Ti could be a good alternative for patients who showed an increased sensitization to Co-Cr alloys. In this study, one patient was first treated with Co-Cr framework for the mandible. The patient reported soreness, burning sensation of mucosa, dryness of the mouth, and redness. Patient treatment was hence rapidly replaced with a pure Ti framework. After a use of 1 week, the patient did not have complaints, and no complications occurred further during the next 2 years. The oral mucosa had no signs of irritation. It was concluded that Ti was preferable to Co-Cr alloys for RPD applications. It is important to note that this was a single-patient case report. It is therefore unlikely that this result can provide sufficiently comprehensive conclusions.

Another investigation conducted by Łukomska-Szymańska et al. [38] revealed that Co-Cr alloys did not have protective qualities for the oral cavity, which is important to consider for high-sensitive patients. They analyzed the effect of titanium nitride (TiN) coatings on Co-Cr alloy in framework dentures on human palatal epithelium cytology. The results were compared to two other groups, namely Co-Cr alloy in framework dentures without TiN coating and acrylic dentures. While each prosthesis disturbed palatal epithelium keratinization, Co-Cr alloys were associated with a significantly higher perturbation of keratinization compared to acrylic dentures. 

Al-Imam et al. [2] argued that not all irritation and sensitization symptoms are caused by Co-Cr release. The authors examined Co release from 84 used (functional) and 32 new (non-functional) prostheses. During the 1–5-year follow-up on 66 patients, unpleasant symptoms were reported. Notable problems were some signs of inflammation of the oral mucosa (in total for 11 participants), oral candidiasis (2 participants), and ill-fitting prosthesis (16 participants). In addition, all 66 participants had insufficient oral hygiene. However, considering that contact allergy was not spotted, inflammation in 11 participants was related to candidiasis, poor oral hygiene, and ill-fitting prosthesis. Functional prostheses did not release Co, while it was released from 24 non-functional prostheses. This suggests that Co release was associated with manufacturing stage and disappeared within 1 to 5 y. The authors stated that dental prostheses might not be the primary factor for Co exposure leading to sensitization. The evidence that none of the functional prostheses released Co aligns with the hypothesis that it was present only during the fabrication stage for non-functional prosthesis.

In conclusion, sensitization and irritation to Co-Cr still remains an open research area. Though some findings do not provide evidence for such immune reactions, there are a few cases suggesting a correlation between Cr-Co and sensitization. The symptoms that might occur are soreness, burning sensation of mucosa, dryness of the mouth, and redness. In addition, Co-Cr alloys do not have protective qualities for the oral cavity, which might be one of the factors for the increased sensitization.

### 3.5. Type IV Hypersensitivity Reaction to Co-Cr Alloys

Allergies are a common symptom associated with cytotoxicity. Kettelarij et al. [49] investigated the amounts of Co, Cr, and Ni on the skin and in the urine of dental technicians (*n* = 13) and in the air of their workspaces. The metal dose on skin was investigated with acid wipe sampling and the air exposure by personal air sampling. Co, Cr, and Ni exposures were observed on skin and through the air after 2 h work. Urine samples were analyzed with inductively coupled plasma mass spectrometry. The Co dose on the skin increased significantly. Co was observed in 10 air samples (0.22–155 μg/m^3^), Cr in 9 (0.43–71 μg/m^3^), and Ni in 4 (0.48–3.7 μg/m^3^). After evaluating the results, it was suggested that Co exposure can cause type IV hypersensitivity reactions, commonly called allergic contact dermatitis. 

Contact dermatitis evidence was provided by Song et al. [35] after examining reactions to Co in cast dental crowns. A 58-year-old patient wearing crowns developed skin irritation on his hands and feet, which was reported to be a palmoplantar pustulosis-like allergy. Other observed symptoms included redness, pustules, vesicles, and scaly erythema on hands and feet, appearing 1 month after Co-Cr dental application use on molar teeth. The symptoms were persistent and lasted 1 year. Patch testing revealed strong reactions to Co chloride, suggesting that it can cause allergic reaction. Symptoms disappeared in 3 weeks after crown removal, which is in accordance with previous hypotheses. 

Though it was concluded that Co exposure could be associated with allergic reactions, the data so far are inconsistent. For example, Al-Imam et al. [2] reached a different conclusion. Together with Co release from used functional and new non-functional prostheses, the authors investigated contact allergy to the alloys. Co release from prostheses was examined with the Co spot test and contact allergy by patch testing. In a 1–5-year study with 66 participants, none of them reported allergic reactions to Co. This study was conducted following previous findings that Co alloys were associated with elicit allergic reactions in Co-allergic patients. However, Al-Imam et al. [2] provided contrary results suggesting that Co prostheses are safe to use in dentistry. 

In conclusion, there are no consistent data for Co-Cr-allergizing properties. Some findings clearly support Co-Cr biocompatibility without any allergic reactions though these are not unequivocal. The evident cases of allergic reactions cannot be overlooked. Data inconsistency may be a good reminder for dental practitioners to be cautious of potential allergies when prescribing Co-Cr applications. 

## 4. Discussion

The toxicological risks of Co-Cr are becoming an increasingly important topic nowadays. The aim of this research is to analyze the biocompatibility of Co-Cr alloys for dental uses in humans, based on work published during the period 1995–2020. After systematic scientific literature analysis, there are notable questions to consider.

Compared to Ni based alloys in vitro, Co-Cr alloys were associated with less reactions and did not increase oxidative stress or cellular toxicity. In vitro and in vivo studies included in the present review provided evidence that Ni alloys might be cytotoxic to the human oral cavity. Ni alloys are associated with decreased cell viability, increased oxidative stress, inflammatory cytokine expression, and cellular toxicity levels. The experiment conducted by Ganbold et al. [48] showed lower cell proliferation and viability for Ni-Cr alloys when compared to Co-Cr alloys. Comăneanu et al. [45] supported the relatively good cytocompatibility of all Ni-Cr (N1, N2, N3) and Co-Cr (C1, C2, C3) alloys, but Co-Cr alloys exhibited better cell adhesion and proliferation than Ni-Cr alloys. In vivo, Co-Cr alloys exhibited worse outcomes when compared to Ti. Consistent evidence supports that Ti alloys exhibit the best results in terms of biocompatibility in humans. Hence, the analyzed alloys can be shown in the following descending order: Ti ≥ Cr / Co > Ni in terms of biocompatibility. 

Ti alloy is commonly considered to be an effective substitute to Co-Cr alloys, but due to its properties, Ti is mostly used in dental implantology. However, when patients reported irritation symptoms with Co-Cr RPD, Ti treatment successfully reduced the negative effects as observed by Könönen et al. [31]. Still, various experiments provided evidence in favor of Ti over Co-Cr and Ni alloys (Martín-Cameán et al. [43], Yu et al. [47], Könönen et al. [31]). For this reason, dental applications of Ti alloys could be prescribed for the patients who usually experience allergic reactions to metal exposure.

Some studies demonstrated potential toxicity of Co-Cr dental alloys. For example, Seldén et al. [32] found that Co-Cr-Mo dental constructions could cause pneumoconiosis in dental technicians working in the laboratories without a local exhaust ventilation equipment. Nowadays, that kind of equipment is mandatory for all laboratories, and we strongly recommend conducting an updated, new investigation. Kim et al. [50] was the only recent in vitro study in disfavor of Co-Cr. They observed that Co-Cr alloy exhibits cytotoxic and inflammatory effects. In particular, they found Co-Cr to be cytotoxic to human gingival fibroblasts and osteoblasts. In addition, it significantly increased ROS production and increased levels of inflammatory mediators (iNOS-derived nitrite oxide and COX-2-derived prostaglandin E2). There is not enough evidence to state that Co-Cr alloys are cytotoxic for every patient, as most in vitro studies have observed no toxicity. Rusu et al. [41] reported no cytotoxicity for Co-Cr alloys. Forster et al. [42], McGinley et al. [39,40] and Comăneanu et al. [45] found that Co-Cr alloys exhibited good cell proliferation, while Gălăţeanu et al. [46] observed a decreased cell toxicity with exposure time. As observed by Katsoulis et al. [34], differences between Co-Cr and Ti alloys were not observed in terms of patient compliance. Supported by Al-Imam et al. [2], dental Co-Cr alloys were not associated with contact allergies to Co. The Co release was present only during the manufacturing stage. The authors suggest that this could be due to high resistance to corrosion and passivation of the alloy. Previous research cases of Co release could be associated with mechanical wear, structure of the alloy, thermal treatment, and crevices.

Nevertheless, previous reports of negative immune reaction should not be overlooked. Al-Imam et al. [2] emphasized that Co release could be also affected by local factors, such as inherited oral mucosa sensitivity, poor oral hygiene, ill-fitting prosthesis, and oral product with fluoride. Therefore, even though Co-Cr alloys dental prosthesis might not be the main source for inflammation, the alloys mechanical properties may be a factor increasing the risk of metal release. Considering the inconsistency of data on the biocompatibility of Co-Cr for human dental applications, further research is still needed.

Co-Cr alloys are often used in all fixed or removable prosthetic constructions both for obvious mechanical and economic reasons, and in most of the cases, it is quite complicated to find an effective alternative. Most of the alternatives to Co do not perform in the same way. When it comes to discussing side effects, Co-Cr is not the only alloy that causes complications. The presence of many substances must also be considered in all scientific studies and the corresponding discussions. Many substances can cause allergic reactions and so on.

Interestingly, it is worth noticing that the current systematic review highlighted a gap between in vivo and in vitro studies investigating Co-Cr influence in dental application. While in vivo studies mainly compared Co-Cr-based materials to Ti-based materials, in vitro studies compare Co-Cr to Ni-based materials. This is also important to note that most in vivo studies observed less favorable behavior for Cr-Co alloys, while in vitro studies have found better cytocompatibility. This suggests of in vitro model of the oral cavity to assess for biocompatibility. Further models more representative of the in vivo oral cavity have to be performed to subject cells to faithful 3D models and stimuli in vitro. Furthermore, results from Table 1 show that several in vivo studies were assigned to a low or very low quality due to inconsistency or imprecisions. This highlights the need to define the term biocompatibility in the context of oral medicine. By defining what we are looking for in terms of biocompatibility, it may help in designing in vitro or in vivo biocompatibility studies. 

## 5. Conclusions

According to various studies, Co-Cr alloys could rarely cause sensitization, irritation, and allergic reactions except for patients being allergic to Co or Cr. Among the reported side effects, soreness, burning sensation of mucosa, dryness of the mouth, and redness were the most common. In a few cases, contact dermatitis and palmoplantar pustulosis were directly associated with Co-Cr dental applications. The finding that Co-Cr alloys did not have protective qualities for the oral cavity could help to explain some patients’ sensitization. As not all patients experienced any of these symptoms, further investigations are still to be performed to understand the causes for these symptoms.

A systematic review of described in vitro and in vivo studies leads to the conclusion that in a descending order, biocompatibility of metal alloys is as follows: Ti > Cr / Co > Ni. In comparison to Ni, Co-Cr alloys exhibit a lower cytotoxicity and a higher cell proliferation and viability. Ti alloys show the best biocompatibility when compared to Ni and Co-Cr alloys. The metal has good resistance to corrosion and displays the lowest risk of allergic reaction and negative immune system responses. This has to be considered knowing that, in general in the included studies, Cr-Co is compared to Ti-based materials in vivo and Ni alloys in vitro.

Thus far, the investigations on biocompatibility and cytotoxicity of Co-Cr alloys for human dental applications have provided inconsistent data. On one hand, one in vitro and one in vivo study reported a correlation to pneumoconiosis, certain cytotoxicity and inflammatory effects, increased ROS production, and levels of inflammatory mediators. On the other hand, most of the in vitro studies did not find cytotoxicity for Co-Cr alloys and rather reported good cell proliferation and adhesion. Considering that data inconsistency complicates an objective evaluation, further specific studies assessing Co-Cr dental alloys’ biocompatibility are urgently needed within the current framework of the new European regulations dedicated to Co-Cr alloys [4]. It is therefore important to develop new experimental approaches in vitro to better guide the protocols used in vivo and thus reduce the difference in the observed results.

## Figures and Tables

**Table 1:** Main characteristics of articles extracted from *Medline*, *Embase*, and *Web of Science* databases (1995–2020).

Author Year	Type Duration	Assessed Criteria	Main Results/Conclusion
Könönen et al. (1995) [31]	In vivo 5 y	*n* = 1 Effects of RPD with Ti on oral cavity (case report).	(a) The effects of RPD with a CoCr framework on oral cavity was soreness and burning of labial mucosa, dryness of the mouth, and soreness and redness of the tissues. Subsequently, the throat dried and felt sore, and vesicles were manifest in the palatal mucosa. The symptoms ceased after removal of the denture; (b) The effects of RPD with a CoCr framework coated by gold on oral cavity: intraoral symptoms, but after 1 y, the gold plating had worn away, and symptoms back; (c) The effects of RPD with a Ti framework on oral cavity. After wearing it for 2 y, no complications were observed.
Seldén et al. (1995) [32]	In vivo	*n* = 37 Effect of Co-Cr-Mo exposure to lung disorders.	The dust from Co-Cr-Mo dental constructions can cause pneumoconiosis. Six patients exhibited radiological parameters associated with alloys. The risk can be reduced with local exhaust ventilation.
Seldén et al. (1996) [33]	In vivo 5 y	*n* = 3 Effect of Co-Cr-Mo exposure to lung disorders (a follow-up study of confirmed pneumoconiosis cases in dental technicians working with the alloys).	Pneumoconiosis is associated with inorganic dusts arising in production of Co-Cr-Mo dental constructions. However, the primary causes of 2 reported cases cannot be reduced to alloys because patients already had lung problems.
Katsoulis et al. (2008) [34]	In vivo 12 months	*n* = 10 Effects of using Ti in RPDs of the Ti6A17Nb-alloy.	After 6 months, significant biological differences were not observed. The Ti6A17Nb-alloy (Girotan L) for RPDs can be regarded as equivalent to RPDs made from Co-Cr-alloy.
Song et al. (2011) [35]	In vivo	*n* = 1 Allergic reaction to Co in cast dental crowns (case report).	58-year-old male patient wearing crowns developed palmoplantar pustulosis in the hands and feet. Symptoms include redness, pustules, vesicles, and scaly erythema on hands and feet. It appeared 1 month after Co-Cr application on molar teeth. Symptoms disappeared with the crown removal, confirming allergic reaction to the material.
Imirzalioglu et al. (2012) [36]	In vitro	Effect of repeated casting of alloys on gingival fibroblast cytotoxicity.	Recasting significantly increased elemental release in Co-Cr and Ni-Cr alloys (*p* < 0.001), but Ni-Cr alloys were associated with higher cytotoxicity, especially after recasting Ni-Cr alloys with 65% surplus metal (significant increase).
Baričević et al. (2012) [37]	In vivo >5 y	*n* = 55 Genotoxicity of Co-Cr-Mo and Ni-Cr alloys when exposed to contact with oral cavity.	Comet assay parameters (tail length and percentage DNA in the tail) were significantly higher in the group wearing prosthodontic appliances: (a) Mean of tail length = 13.13 for control group and = 15.85 for experimental group. (b) Main of percentage DNA in the tail = 0.36 for control group and = 2.07 for experimental group.
Łukomska-Szymańska et al. (2012) [38]	In vivo >5 y	*n* = 120 Effect of TiN coatings on Co-Cr alloy in framework dentures on human palatal epithelium cytology in comparison to: (a) Framework dentures without TiN coating; (b) Acrylic dentures.	Co-Cr alloys did not have protective qualities for the oral cavity. Each prosthesis disturbed palatal epithelium keratinization, but Co-Cr alloys were associated with significantly higher perturbation of keratinization in comparison to acrylic dentures.
McGinley et al. (2012) [39]	In vitro 72 h	Ni-Cr alloys cytocompatibility and effect on human-derived oral mucosa.	Co-Cr had significantly better cytocompatibility than Ni-Cr alloy. Ni-Cr alloy-treated oral mucosal models were associated with (i) significant reductions in cell viability and (ii) significant increases in oxidative stress, inflammatory cytokine expression, and cellular toxicity (in comparison to untreated oral mucosal models). The higher the Ni, the higher the effects.
McGinley et al. (2013) [40]	In vitro 72 h	Cytocompatibility of base-metal dental casting alloys (Ni-Cr and Co-Cr) in fixed prosthodontic and orthodontic dentistry.	Ni-Cr base-metal alloy immersion solutions shown significantly lower cytocompatibility than Co-Cr alloys. In comparison to controls, Ni alloy was associated with significantly decreased cell viability, increased oxidative stress, inflammatory cytokine expression, and cellular toxicity levels. Co-Cr alloy did not increase oxidative stress or cellular toxicity when compared to controls.
Rusu et al. (2014) [41]	In vitro 7 days	Cytotoxicity of Ni-Cr and Co-Cr alloys.	The cytotoxicity of both alloys was similar, suggesting non-cytotoxic effect. After 7 days of inoculation, the cells grew well for both alloys and had a relatively high confluence. They observed no fragments detached with the eluates.
Forster et al. (2014) [42]	In vitro 72 h	Attachment and proliferation rate of cultured human epithelial cells on these materials: (a) Polished lithium (Li)-disilicate; (b) Yttrium-modified zirconium dioxide; (c) Co-Cr alloy.	All surfaces exhibited significant cell proliferation in comparison to control plate (Li-disilicate, zirconia, Co-Cr). Li-disilicate exhibited the highest cell attachment and zirconia the lowest. It revealed that all restorative materials were equally suitable for subgingival restorations, but Li-disilicate had the best cytocompatibility.
Martín-Cameán et al. (2015) [43]	In vivo 15 months	*n* = 60 Determination of the content of metals (Al, Cu, Cr, Mn, Ni, Ti, and V) in oral mucosa cells from patients treated with conventional orthodontic appliances (brackets, arch wires, and bands) in comparison to: (a) Patients treated additionally with mini-screws; (b) Control group.	Only few traces of Co and V release were observed. The rest can be summarized in such order: Cr < Ni < Ti < Cu < Al. Significant differences in metal release compared to the control group were observed: (a) For Ni (for orthodontic and orthodontic + mini-screw groups); (b) For Cu (for orthodontic group).
Puskar et al. (2015) [44]	In vitro	Cytotoxicity of DMLS and cast Co-Cr-Mo dental alloy on human MRC-5 fibroblast cells.	Corresponding alloy did not have negative cytotoxic effect and could be used for application in dentistry. Cytotoxic effect was observed in neither conventionally cast nor DMLS Co-Cr-Mo alloy. There was no statistically significant difference between samples.
Comăneanu et al. (2015) [45]	In vitro	Cytocompatibility of Ni-Cr (N1, N2, N3) and Co-Cr (C1, C2, C3) alloys.	Cytocompatibility of the alloys examined can be summarized in the following descending order: C1 > C3 > N2 > N3 > C2 > N1. Co-Cr alloys were associated with better cell adhesion.
Al-Imam et al. (2016) [2]	In vivo 1–5 y	*n* = 66 (a) Co release from 84 used (functional) and 32 new (non-functional) prostheses; (b) Contact allergy.	(a) Used prostheses did not release Co, while it was released in 24 new prostheses. It was revealed that Co release was associated with manufacturing stage and disappeared within 1–5 y. (b) Contact allergy was not spotted, and inflammation in 11 participants was related to candidiasis, poor oral hygiene, and ill-fitting prosthesis.
Gălăţeanu et al. (2016) [46]	In vitro 24 h	Electrochemical behavior of two Co-Cr dental alloys: (1) Wirobond 280; (2) Wirobond C (with some % of Ga and Mn).	Wirobond 280 exhibits best qualities: (a) Correlated with the greater cell viability; (b) Correlated with smaller level of intracellular ROS.
Kettelarij et al. (2016) [25]	In vivo 24 h	*n* = 13 Co, Cr, and Ni exposure on the skin, in the air, and urine levels for dental technicians.	Co, Cr, and Ni exposure after work (2 h) were observed on skin and through the air: (a) Co dose on the skin after work increased significantly; (b) Co was observed in 10 air samples (0.22–155 μg/m^3^), Cr in 9 (0.43–71 μg/m^3^), and Ni in 4 (0.48–3.7 μg/m^3^). (c) It was concluded that Co exposure could be associated with allergic contact dermatitis and sensitization.
Kim et al. (2016) [24]	In vitro 24 h	Effects of Co-Cr alloys on HGF and osteoblasts.	Few Co-Cr alloy cytotoxic and inflammatory effects via activation of Nrf2/ARE were examined: (a) It was revealed to be cytotoxic to HGFs and osteoblasts; (b) Significantly increased ROS production; (c) Upregulated pro-inflammatory cytokines; (d) Increased levels of inflammatory mediators (iNOS-derived nitrite oxide and COX-2-derived prostaglandin E2).
Yu et al. (2017) [47]	In vivo 12 months	*n* = 196 The peri-implant clinical parameters (PI) and the concentrations of RANKL, OPG, and calcium in PICF with four different crown materials (Co-Cr, Au-Pt, Ti, Zi).	All materials affected the concentrations of OPG, RANKL, calcium ion, and RANKL/OPG ratio. 12 months after restoration, PD and GCF volumes for all groups were significantly higher when compared to control group. Zi and Ti had the best parameters. Ti group had the highest OPG concentration; Ti and Zi groups had smallest concentrations of RANKL and calcium ion, as well as smallest RANKL/OPG ratio.
Ganbold et al. (2019) [48]	In vitro	hADSC behavior on a 3D printed Co-Cr alloy in comparison to other Co-Cr alloys (made by casting or milling) and Ni-Cr alloy.	Ni-Cr alloy was associated with significantly lower cell proliferation and viability. OD values for all Co-Cr groups (casting, milling, and 3D) were higher than that of the Ni-Cr group. It reveals that all Co-Cr alloys are more cytocompatible than Ni-Cr alloy.

**Table 2:** GRADE assessment for in vivo studies. Overall quality: ++++ high, +++ moderate, ++ low, + very low.

Author	Study Limitations	Inconsistency	Indirectness	Imprecision	Publication Bias	Overall Quality
Könönen (1995) [31]	V	V	V	**X**	V	+++
Seldén (1995) [32]	V	**XX**	V	V	V	++
Seldén (1996) [33]	V	**XX**	V	**X**	V	+
Katsoulis (2008) [34]	**X**	V	V	**X**	V	++
Song (2011) [35]	V	V	V	**X**	V	+++
Łukomska-Szymańska (2012) [38]	V	V	**X**	V	V	+++
Baričević (2012) [37]	V	V	**X**	V	V	+++
Martín-Cameán (2015) [43]	V	V	**X**	V	V	+++
Kettelarij (2016) [49]	V	**XX**	V	**X**	V	+
Al-Imam (2016) [2]	V	V	V	V	V	++++
Yu (2017) [47]	V	**X**	**X**	V	V	++

V, no downgrading; X, one-point downgrading; XX, two-point downgrading.

**Table 3:** GRADE assessment for in vitro studies. Overall quality: ++++ high, +++ moderate, ++ low, + very low.

Author	Study Limitations	Inconsistency	Indirectness	Imprecision	Publication Bias	Overall Quality
McGinley (2012) [39]	V	V	V	V	V	++++
Imirzalioglu (2012) [36]	V	V	V	V	V	++++
McGinley (2013) [40]	V	V	V	V	V	++++
Rusu (2014) [41]	**X**	V	V	**X**	V	++
Forster(2014) [42]	V	V	V	**X**	V	+++
Puskar (2015) [44]	V	V	**X**	**X**	V	++
Com*ă*neanu (2015) [45]	**X**	V	V	**X**	V	++
Gălăţeanu (2016) [46]	V	V	**X**	V	V	+++
Kim (2016) [24]	V	**X**	**X**	V	V	++
Ganbold(2019) [48]	V	V	V	V	V	++++

V, no downgrading; X, one-point downgrading; XX, two-point downgrading.
